# Phosphorus Deficiency Inhibits Cell Division But Not Growth in the Dinoflagellate *Amphidinium carterae*

**DOI:** 10.3389/fmicb.2016.00826

**Published:** 2016-06-01

**Authors:** Meizhen Li, Xinguo Shi, Chentao Guo, Senjie Lin

**Affiliations:** ^1^State Key Laboratory of Marine Environmental Science and Marine Biodiversity and Global Change Research Center, Xiamen UniversityXiamen, China; ^2^Department of Marine Sciences, University of Connecticut, GrotonCT, USA

**Keywords:** *Amphidinium carterae*, phosphorus deficiency, cell size, cell cycle, Rubisco

## Abstract

Phosphorus (P) is an essential nutrient element for the growth of phytoplankton. How P deficiency affects population growth and the cell division cycle in dinoflagellates has only been studied in some species, and how it affects photosynthesis and cell growth remains poorly understood. In the present study, we investigated the impact of P deficiency on the cell division cycle, the abundance of the carbon-fixing enzyme Rubisco, and other cellular characteristics in the Gymnodiniales peridinin-plastid species *Amphidinium carterae*. We found that under P-replete condition, the cell cycle actively progressed in the culture in a 24-h diel cycle with daily growth rates markedly higher than the P-deficient cultures, in which cells were arrested in the G1 phase and cell size significantly enlarged. The results suggest that, as in previously studied dinoflagellates, P deficiency likely disenables *A. carterae* to complete DNA duplication or check-point protein phosphorylation. We further found that under P-deficient condition, overall photosystem II quantum efficiency (*F*v/*F*m ratio) and Rubisco abundance decreased but not significantly, while cellular contents of carbon, nitrogen, and proteins increased significantly. These observations indicated that under P-deficiency, this dinoflagellate was able to continue photosynthesis and carbon fixation, such that proteins and photosynthetically fixed carbon could accumulate resulting in continued cell growth in the absence of division. This is likely an adaptive strategy thereby P-limited cells can be ready to resume the cell division cycle upon resupply of phosphorus.

## Introduction

Phosphorus (P) plays an essential role in cellular structure and function of living organisms because it is required in vital molecules such as nucleic acids (DNA, RNA), phospholipids (membrane constituents), inositol triphosphate (signaling molecule), reduced nicotinamide adenine dinucleotide (NADH) and its phosphorylated form (NADPH; both reducing equivalents), and adenosine triphosphate (ATP; energy currency). Besides, many enzymes critical in major metabolic pathways and cell division cycle progression are activated by phosphorylation. Thus, P availability is expected to impact the survival and vitality of marine phytoplankton and hence influence marine primary production. In the euphotic zone P is rapidly consumed for photosynthesis and resupply (mainly from recycling in the open ocean or upwelling and terrestrial discharge in the coastal zone) is slow. As such, the preferred form of P, dissolved inorganic phosphorus (DIP), often falls below growth-limiting concentrations in many parts of the ocean, including oligotrophic oceans and some coastal waters (Smith, 1984; Wu et al., 2000; Thingstad et al., 2005; Sylvan et al., 2006; for reviews see Karl, 2014 and Lin et al., 2015). Phytoplankton have evolved adaptive mechanisms to cope with the shortage of DIP (Lin et al., 2015), e.g., enhancing ability to take up low-abundance DIP by means of high affinity phosphate transporters (Orchard et al., 2009), scavenging P from dissolved organic phosphorus via the action of alkaline phosphatase and other hydrolytic enzymes (Benitez-Nelson and Buesseler, 1999; Reynolds et al., 2014), and decreasing phosphorus demand by substituting phospholipids with sulfolipids (Van Mooy et al., 2006, 2009) or accelerating phospholipid turnover to provide short term P supply (Martin et al., 2011).

One common effect of P deficiency among phytoplankton species is inhibition of population growth. As population growth results directly from the completion of the cell cycle, information on how P limitation affects the cell cycle would be insightful for understanding the mechanism underlying the growth inhibiting effect of P deficiency. A typical cell cycle is divided into four distinct phases, the S phase (DNA synthesis), M phase (nucleus division), and two gaps between G1 (growth stage before S phase) and G2 (preparatory stage before mitosis/cytokinesis) stages. P limitation has been observed to cause lengthened G1 phase in the cyanobacterium *Synechococcus* (Vaulot et al., 1996), and P starvation caused cell cycle arrest at any phase (including unrecoverable arrest at S) in *Prochlorococcus* spp. (Parpais et al., 1996). Information regarding cell cycle effects of P limitation on eukaryotic phytoplankton is limited and largely restricted to few dinoflagellate species. Studies so far indicate that P limitation causes G1 phase arrest in dinoflagellates, including the Gymnodiniales fucoxanthin-plastid species *Karenia mikimotoi* (Lei and Lu, 2011), the Gonyaulacales species *Alexandrium pacificum* (formerly *A. catenella*; Zhang et al., 2014), and the Prorocentrales species *Prorocentrum donghaiense* (Li et al., 2015), all of which cause harmful algae blooms (HABs). More dinoflagellate species need to be examined to understand if such cell cycle responses to P limitation are universal in the phylum of Dinoflagellata. Besides, the previous studies consistently indicated that while arrested in G1 phase under P limitation cells became enlarged, raising a question whether photosynthetic carbon fixation and protein synthesis persisted during P limitation. Furthermore, suppression in photosynthetic efficiency in photosystem (PS) II has been reported under P limitation in some phytoplankton species, including the diatoms *Phaeodactylum tricornutum* (Lin et al., 2013) and *Thalassirosira weissflogii* (Liu et al., 2011) and the green algae *Sphaerocystis*, *Scenedesmus* (Beardall et al., 2001) and *Dunaliella tertiolecta* (Geider et al., 1998). However, different responses to P limitation have been reported on Ribulose 1, 5-bisphosphate carboxylase/oxygenase (Rubisco), the key enzyme for CO_2_ fixation in the Calvin-Benson-Bassham pathway. P limitation was found to decrease Rubisco abundance in the marine diatom *Skeletonema costatum* (Liu et al., 2013) and the Coccolithophyceae *Emiliania huxleyi* (Losh et al., 2013) whereas in the marine diatom *P. tricornutum* Rubisco seemed unaltered or up-regulated in oligotrophic conditions (Geider et al., 1993; Feng et al., 2015). However, how P deficiency impacts these indicators of photosynthesis capability has remained unexplored in dinoflagellates.

In this research, using *Amphidinium carterae* as a model, we aimed at gaining understanding on how P deficiency impacts the cell cycle, photosynthesis capability, and cellular features such as cellular contents of carbon and nitrogen as well as cell size. *A. carterae* represents a Gymnodiniales species that nevertheless contains peridinin as the major accessory pigment (while most species in this class contains fucoxanthin instead) and a harmful algal bloom species (Hallegraeff, 1993; Hofmann et al., 1996). We found that under P limitation, cells of this species were arrested in the G1 phase, yet enlarged in size, slightly but not significantly reduced in PSII efficiency and Rubisco abundance, and higher in C and N contents, which suggests an adaptive strategy to remain ready to resume cell division upon restoration of P supply.

## Materials and Methods

### Algal Culture and Phosphorus Treatments

*Amphidinium carterae* strain CCMP1314 (provided by Provasoli-Guillard National Center for Marine Algae and Microbiota) was cultured at 20 ± 1°C under a 14 h/10 h light/dark cycle with a photon flux of 100 μmol⋅m^-2^⋅s^-1^. L1 growth media (without silicate) were prepared with varying P concentrations using autoclaved oceanic water collected from South China Sea, where soluble reactive phosphorus was below the detection limit (0.01 μM) of the Phosphomolybdenum Blue Spectrophotometric Method (Parsons, 1984). For the P-deprived experimental group, the L1 medium was prepared with a reduced phosphate concentration (10 μM), which was consumed until 1 μM before the start of the experiment (designated as day 1). Thereafter, no more phosphate was added during the experiment. A P-replete treatment group was set up as the control, in which 36 μM NaH_2_PO_4_ was added. Both the control and the treatment were triplicated. Unless specified otherwise, all experimental cultures were grown in the volume of 4.5 L in 5-L flasks.

### Measurement of Cell Concentration, Population Growth Rate, Cell Size, and DIP Concentration

Since day 1, samples were collected daily from each of the cultures for physiological parameter analyses. Cells were fixed in Lugol’s solution, cell counts were obtained microscopically with Sedgewick-Rafter counting chamber (Lin et al., 2012), and daily population growth rate was calculated as μ = ln(*N*_2_/*N*_1_)/(*t*_2_-*t*_1_), where *N*_2_ and *N*_1_ are cell concentrations on day *t*_2_ and day *t*_1_, respectively. Except in the diel sample set which was analyzed using flow cytometer (see below), the mean cell size was measured as equivalent spherical diameter using Z2-Coulter^®^ Particle Counter (Beckman Coulter, USA), with a particle size range of 5–15 μm. From each culture a 25-mL sample was filtered through 0.22-μm mixed-cellulose-ester membrane. The DIP in the filtrate was determined using the Phosphomolybdenum Blue Spectrophotometric Method (Parsons, 1984) on 722 Visible Spectrophotometer (CANY, China).

### Measurement of Chlorophyll *a* and Photochemical Efficiency (*F*v/*F*m Ratio)

A 25-mL sample from each culture was filtered onto a 25 mm GF/F filter. The filter was immersed in 90% acetone and kept at 4°C in the dark for 48 h to extract Chlorophyll *a*, which was measured using Turner Trilogy (Turner Designs fluorometer, USA) following the JGOFS protocol (Ducklow and Dickson, 1994a) and averaged to per cell content. Photochemical efficiency was quantified with Xe-PAM (Walz, Germany) after the samples were kept in darkness for 15–30 min.

### Measurement of Cellular Carbon and Nitrogen Contents and Protein Concentration

Every time a sample was taken for cell count and DIP measurement, a 25-mL sample from each culture was filtered onto a 25-mm GF/F filter which had been pre-combusted at 450°C for 5 h in a Muffle Furnace. The cell-containing filter was combusted, and C and N were measured in PE2400 SERIESII CHNS/O Elemental Analyzer (Perkin Elmer, USA) following the JGOFS protocol (Ducklow and Dickson, 1994b), and the weight of each element was averaged to per cell content.

Cells for protein concentration analysis were harvested from 200-mL samples by centrifugation (5000 × *g*, 10 min, 4°C) and homogenized in 1 × PBS (phosphate buffered saline, PH = 7.4) by bead-beating (0.5 mm diameter ceramic beads) on FastPrep^®^-24 Sample Preparation System (MP Biomedicals, USA). The homogenate was centrifuged at 12,000 × *g*, for 2 min, and the supernatant was collected in a fresh tube. Protein concentration was measured using BCA Protein Assay Kit (TianGen Biotech, China) with 30-min incubation at 37°C on SpectraMax^®^ Paradigm^®^ microplate reader (Molecular Devices, USA) at 562-nm wavelength. A dilution series of bovine serum albumin was used as the standard. The data were averaged to per cell content.

### Culture Synchronization and Flow Cytometric Analysis of the Cell Cycle

A culture grown in L1 medium with reduced DIP (10 μM) to be used in the experiment was first synchronized by darkness induction, as previously reported (Taroncher-Oldenburg et al., 1997; Figueroa et al., 2007; Shi et al., 2013). Briefly, culture growing in the exponential phase was removed into continuous darkness for 48 h, and subsequently returned into the normal 14 h/10 h light/dark cycle. Four days later, this synchronized culture was used to set up a new experiment, which consisted of triplicated P-replete and P-deprived cultures, each in 4.5-L in a 5-L culture flask. When the P-replete group indicated exponential growth on day 3, 50-mL samples were collected from each culture every 2 h for flow cytometric cell cycle analysis over a 24-h period. Meanwhile, 200-mL samples were collected every 4 h for subsequent protein concentration and Western blot (for Rubisco) analyses. Cells for cell cycle analysis were harvested by centrifugation (5000 × *g*, 10 min, 4°C) and fixed in 1 mL 70% ethanol solution. Subsequent steps were carried out essentially following Li et al. (2015). Briefly, fixed samples were centrifuged (2000 × *g*, 10 min, 4°C) to pellet the cells, which were washed with 1 × PBS (phosphate buffered saline, pH = 7.4). The cells were then re-suspended in 1 mL ice cold absolute methanol and kept at 4°C for 24 h to extract pigments whose autofluorescence would otherwise interfere with DNA fluorescence measurement. Then samples were stained with propidium iodide (PI; Sigma, St. Louis, MO, USA; 10 μg mL^-1^ prepared in 0.5 mL PI-PBS solution) containing 0.1 mg mL^-1^ RNase (to digest RNA) in darkness overnight at room temperature.

Cell cycle analysis of the PI-stained cells was implemented on CytoFLEX flow cytometer (Beckman Coulter, USA) with the excitation light of 488 nm and detected emission at 620 nm, with signals from 30,000 randomly encountered cells collected for each sample. Based on the flow cytometric data, the cell cycle profile in each sample was analyzed using ModfitLT-1 software. The mean value of the forward scatter (FSC-A) of the cells was taken as relative cell size.

### Analysis of Rubisco Abundance with Western Blot

Protein samples were mixed with Laemmli buffer and denaturized at 95°C for 5 min. For each sample, 20 μg proteins for Rubisco analysis and 30 μg for internal reference (glyceraldehyde-3-phosphate dehydrogenase or GAPDH) analysis were loaded in 10% SDS-PAGE gels (Bio-Rad, USA). Electrophoresis was run at 90 V for 30 min and then at 120 V for 45 min. The resolved proteins were transferred to a polyvinylidene difluoride (PVDF) membrane (Immun-Blot^®^ PVDF Membrane for Protein Blotting, Cat. #162-0177, Bio-Rad, USA) using Trans-Blot SD Semi-Dry Transfer Cell (Bio-Rad, USA) at 25 V for 30 min. After the transfer, the membranes were blocked in 5% defatted milk–TBST solution [Tris buffered saline (TBS) containing 0.1% Tween-20] at 4°C overnight, and then incubated for 1 h at room temperature, respectively, with dinoflagellate Form II RuBisCO antiserum (Zhang and Lin, 2003) at 2500-fold dilution in TBST and GAPDH antiserum (Cat NO. D261392, BBI Life Science, Sangon Biotech, Shanghai, China) at 2500-fold dilution in TBST. GAPDH was used for an internal reference in this research because its abundance had been shown to be less variable than other proteins examined in our laboratory (Shi et al., 2015). Previous studies have shown that housekeeping genes or proteins that are appropriate reference for normalizing mRNA or protein abundances vary among species and environmental variables (e.g., Rosic et al., 2011; Ji et al., 2015). Similar to our own test result cited above, GAPDH has also been shown to be an appropriate reference in some other organisms (e.g., Barber et al., 2005; Goossens et al., 2005). The membranes were then washed with TBST twice and TBS twice, 10 min each. Next, the membranes were incubated in biotinylated goat anti-rabbit IgG (TransGen Biotech, Beijing, China) in 2500-fold dilution for 1 h at room temperature and washed with TBST twice and TBS twice, 10 min each. Finally, the membranes were treated with the enhanced chemiluminescent (ECL) substrate (Bio-Rad, Hercules, CA, USA) for 5 min at room temperature to detect the immunoreactive bands and the abundance of the two proteins were quantified from the band intensities using Image Lab^TM^ software, Molecular Imager^®^ Chemi Doc XR system (Bio-Rad, Hercules, CA, USA).

### Statistical Analysis

In order to evaluate the statistical significance of the differences observed between two treatments, analysis of variance (ANOVA) was carried out using PASW Statistics 18 software package. All data presented are means with standard deviation calculated from the triplicated cultures in each P condition.

## Results

### Culture Growth and DIP Concentration

**FIGURE 1:**
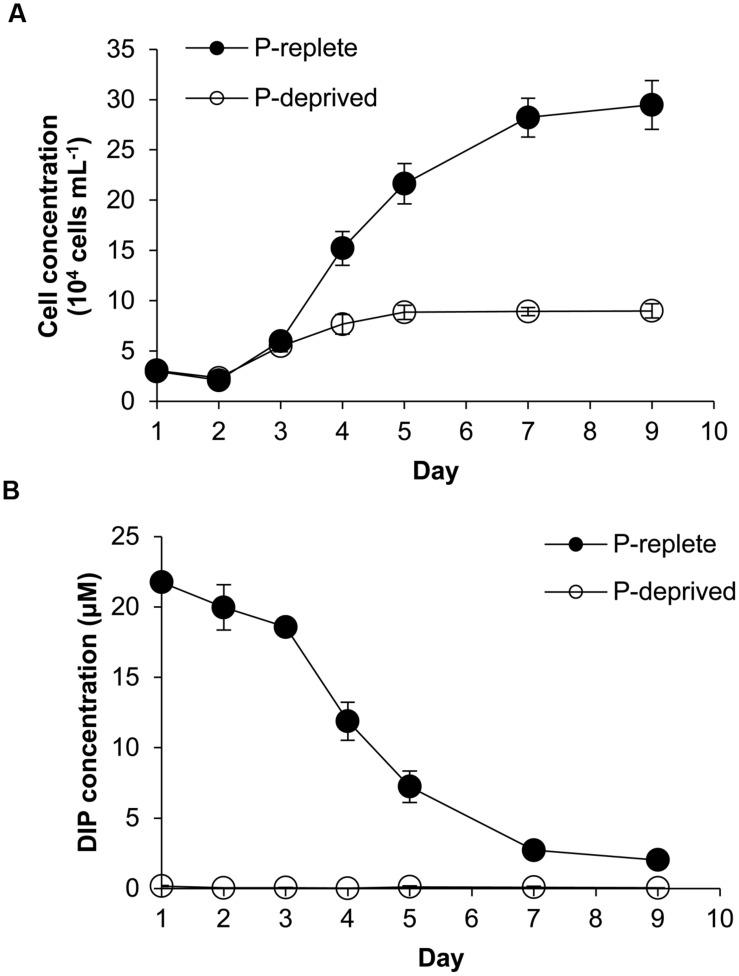
**Growth curves **(A)** and dissolved inorganic phosphorus (DIP) concentrations **(B)** in the *Amphidinium carterae* cultures under the P-replete and P-deprived conditions in the 9-day experimental period.** Shown are means ± standard deviations (error bars) from the triplicated cultures.

Despite similar initial cell densities, cell concentrations in the two P treatment groups started to diverge in 3 days (**Figure 1A**). Cells of the P-replete group maintained exponential growth from day 2 to day 7 with an average growth rate of 0.52 day^-1^ and maximum biomass of ∼290,000 cells mL^-1^ in the 9-day experimental period. In contrast, cell concentrations in the P-deprived group reached a plateau in 4 days with the much lower maximum biomass at ∼80,000 cells mL^-1^ and an average growth rate 0.27 day^-1^ during the experimental period.

With the growth of cell population, DIP concentration in the culture medium of the P-replete group decreased rapidly until it reached about 2 μmol L^-1^ (**Figure 1B**). In the P-deprived group, DIP concentration in the growth medium was consistently undetectable throughout the experiment (**Figure 1B**).

### Cell Size, Cellular Protein, Organic Carbon, and Nitrogen

**FIGURE 2:**
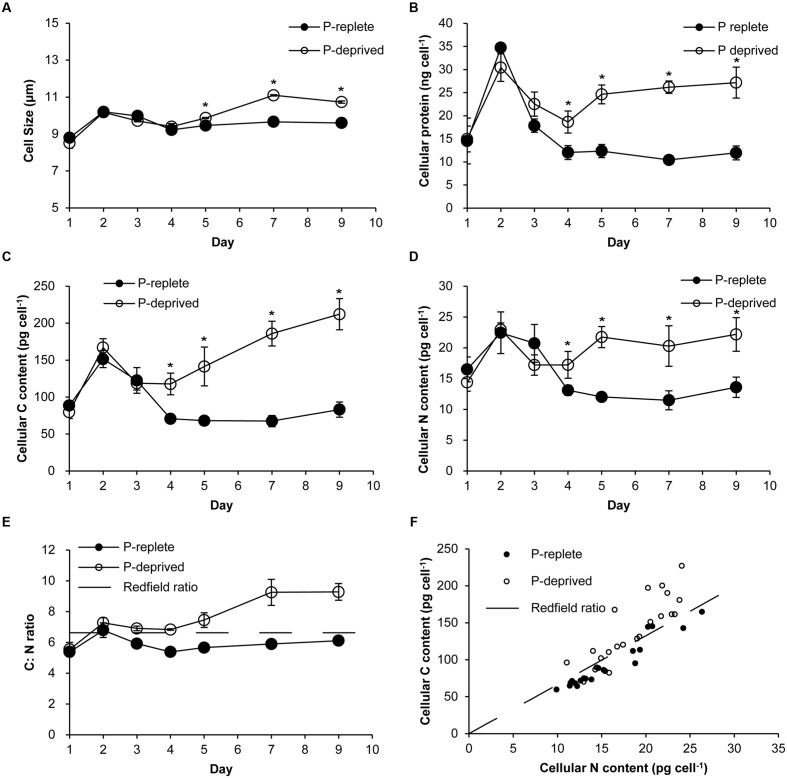
**Cellular parameters of *A. carterae* grown under the P-replete and P-deprived conditions.** **(A)** cell sizes; **(B)** cellular protein content; **(C)** cellular carbon content; **(D)** cellular nitrogen content; **(E)** cellular C: N ratio; the dashed line depicts Redfield C: N ratio; **(F)** correlation between carbon and nitrogen contents; the dashed line depicts Redfield C: N ratio. Except in **(F)**, data shown are means ± standard deviations (error bars) from the triplicated cultures. Asterisks represent that significant differences between P-replete and P-deprived conditions were detected (*p* < 0.05).

Cell sizes were similar between the two treatment groups in the first 4 days of the experiment (**Figure 2A**). In the P-replete group, cell size and cellular contents of proteins (**Figure 2B**), carbon (**Figure 2C**), and nitrogen (**Figure 2D**) increased in the first 2 days and then dropped as a result of active cell division. Contrastingly, these cellular parameters in the P-deprived group kept increasing after day 4, exceeding the cells in the P-replete group gradually. From day 4 on, the averaged carbon, nitrogen, and protein contents per cell in the P-deprived group were twice as much as in the P-replete group, with the differences all being statistically significant (**Figures 2B–D**; *p* < 0.05). The increase in C exceeded that in N (**Figures 2E,F**).

### Cellular Chlorophyll *a* and the Photochemical Efficiency of PSII (*F*v/*F*m Ratio)

**FIGURE 3:**
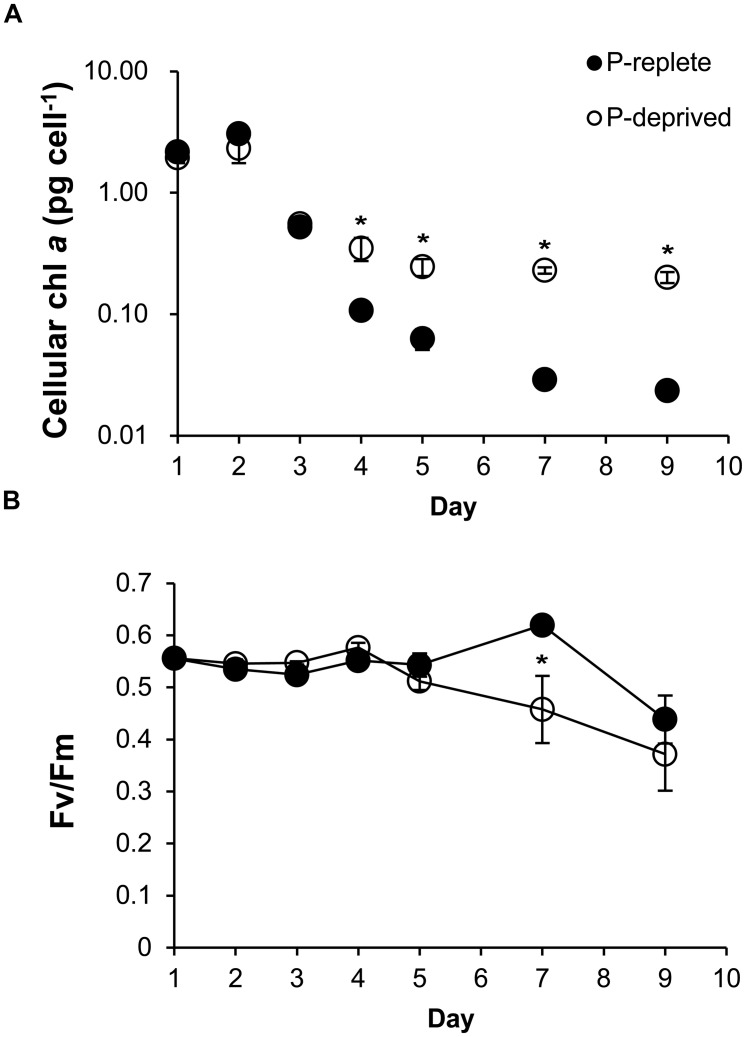
**Cellular chlorophyll *a***(A)** and the maximum photochemical efficiency of PSII (*F*v/*F*m; **B)** of *A. carterae* under P-replete and P-deprived conditions.** Shown are means ± standard deviations (error bars) from the triplicated cultures. Asterisks represent that significant differences between P-replete and P-deprived conditions were detected (*p* < 0.05).

Cellular chlorophyll *a* started to be higher in the P-deprived group than in the P-replete group on day 4 (*p* < 0.05), and the difference increased over time (**Figure 3A**). Photochemical efficiency of PSII (*F*v/*F*m ratio) did not show significant difference between the two P treatment groups throughout the experimental period (*p*> 0.05) except on day 7 (**Figure 3B**). As a result, overall the *F*v/*F*m ratio was not significantly lower in the P-deprived group than in the P-replete group.

### Diel Cell Cycle Patterns of *A. carterae* under P-replete and P-deprived Conditions

**FIGURE 4:**
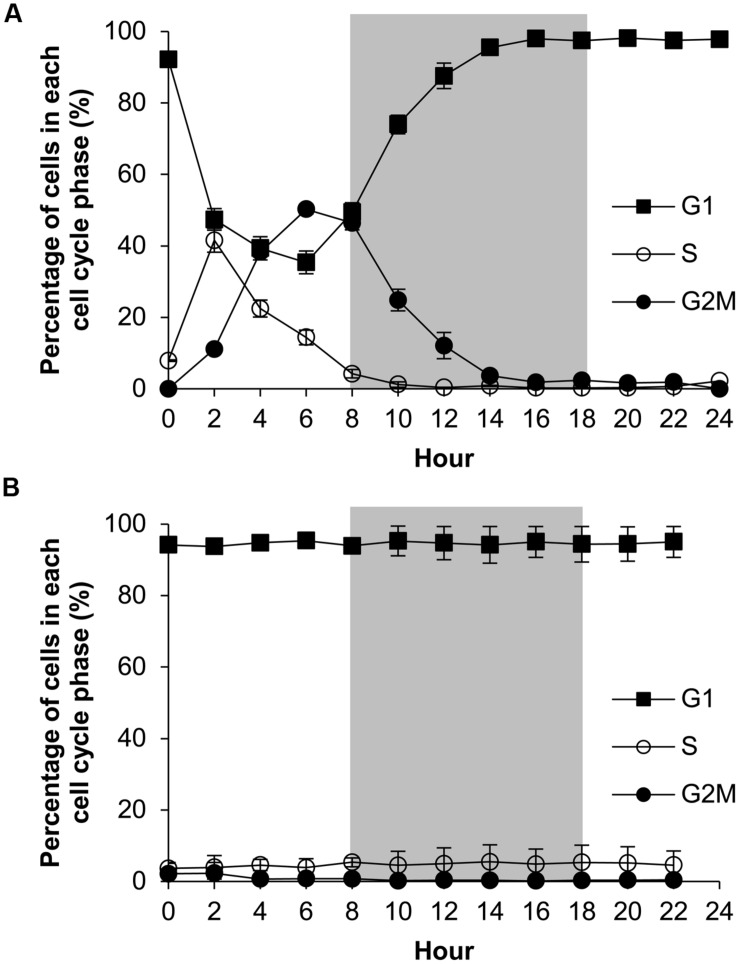
**Diel cell cycle profiles of *A. carterae* in the P-replete group **(A)** and P-deprived group **(B)**.** Gray shading indicates dark period. Shown are means ± standard deviations (error bars) from the triplicated cultures.

**FIGURE 5:**
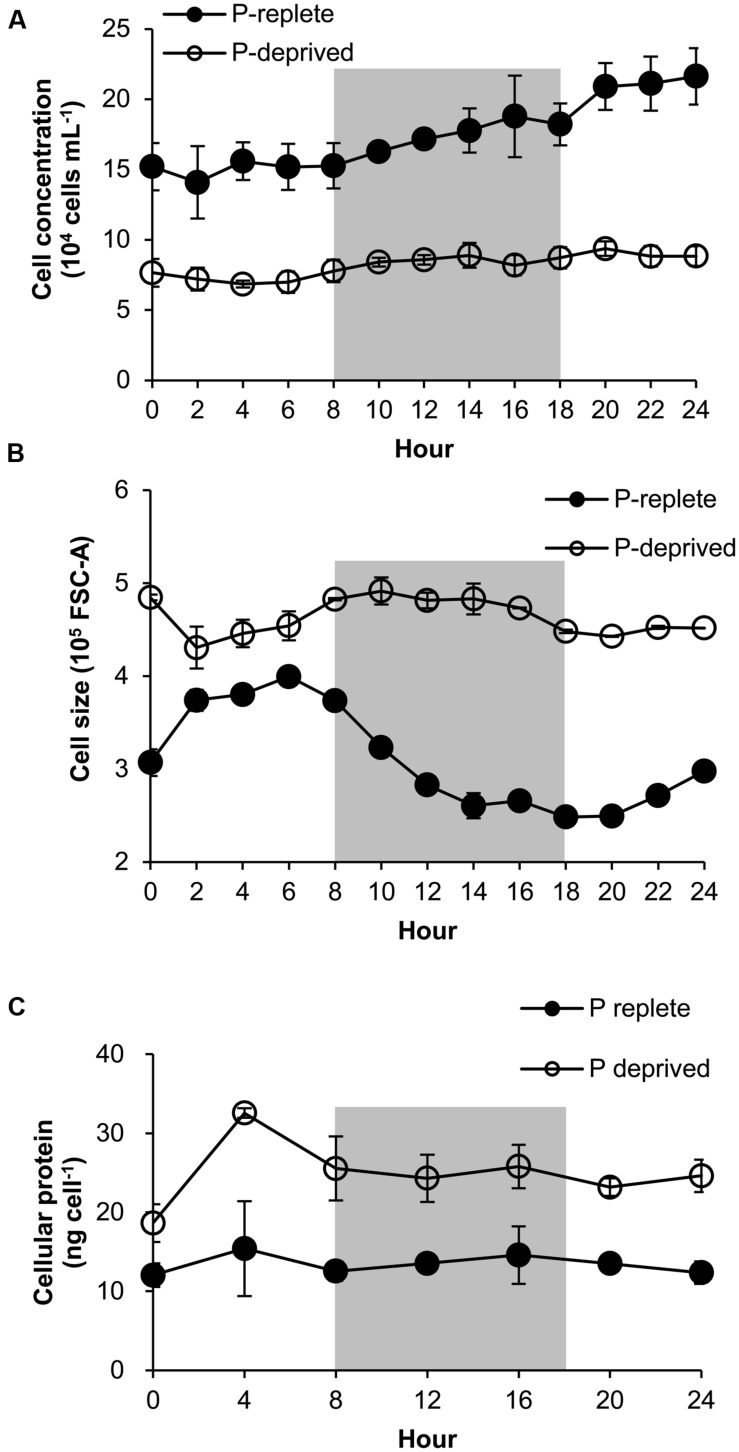
**Growth curve **(A)**, cell size **(B)**, and protein content **(C)** of *A. carterae* in a 24-h diel cell cycle.** Cell size values in **(B)** are forward scatter values as proxies. Gray shading indicates dark period. Shown are means ± standard deviations (error bars) from the triplicated cultures. Statistically significant difference was found between the two P treatment groups in all cases (*p* < 0.05).

The P-replete group showed active cell cycle progression (**Figure 4**, **Supplementary Figure S2**). Cells started to synthesize DNA (S phase) from h0 (**Figure 4A**) and G1 cells decreased from ∼90 to ∼45%, after which the cell size increased (**Figure 5B**). G2M peaked at dusk (h6, 2 h before light turn-off) and decreased quickly to nearly 0 after the middle of the dark period, coinciding with cell concentration increase and sharp cell size decrease (**Figures 5A,B**) and fast increase in G1 cells (**Figure 4A**), indicating active cytokinesis (cell division). The cellular protein content during the 24-h diel cell cycle varied little (**Figure 5C**). Notably, the cells of the P-replete group synthesized DNA in the light period (peaking at h2, middle of the light period).

The P-deprived group, on the contrary, showed elevated size and protein concentration, and lower growth rate (**Figure 5A**) compared with the P-replete group (*p* < 0.05; **Figures 5B,C**). In the 24-h diel cycle, the S and G2M phases were hardly detectable by flow cytometry and over 90% cells was in G1 phase constantly (**Figure 4B**), indicating cell cycle arrest in the G1 phase.

### Rubisco Abundance of *A. carterae* under P-replete and P-deprived Conditions

**FIGURE 6:**
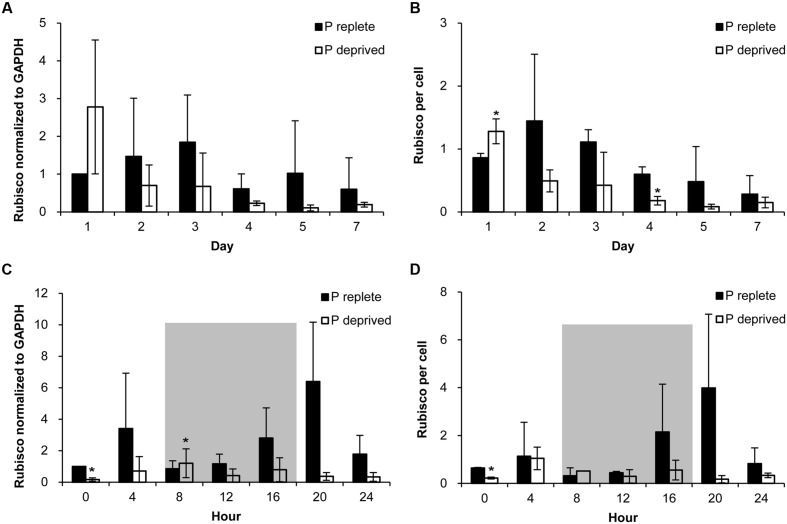
**Abundance of Rubisco in *A. carterae* in the P-replete group and P-deprived group normalized to GAPDH and to per cell measured daily over the 7-day observation period **(A,B)** and every 4 h over a 24-h diel cycle **(C,D)**.** Gray shading indicates dark period. Shown are means ± standard deviations (error bars) from the triplicated cultures. Asterisks represent that significant differences between P-replete and P-deprived conditions were detected (*p* < 0.05).

Rubisco abundance, normalized to GAPDH or to per cell basis, appeared to be higher in the P-replete group than in the P-deprived group except on day 1 (**Figures 6A,B**; **Table 1**). However, considerable variations were observed among the triplicates in each of the treatment group, rendering the differences not statistically different in most of the sample sets.

**Table 1:** Specific growth rates (μ, day^-1^) and comparison (ratios) of Rubisco abundance (normalized to GAPDH and per cell) between P-replete and P-deprived conditions in the 7-day experimental period (mean ± standard deviation).

Day	Growth rate		Rubisco/GAPDH	Rubisco per cell
			
	P-replete	P-deprived	P-replete/P-deprived	P-replete/P-deprived
1	-0.35 ± 0.05	-0.28 ± 0.12	0.43 ± 0.28	0.68 ± 0.05*
2	1.06 ± 0.08	0.86 ± 0.15	1.89 ± 0.89	2.68 ± 1.48
3	0.93 ± 0.13	0.34 ± 0.19	6.34 ± 7.51	7.10 ± 5.45
4	0.35 ± 0.17	0.14 ± 0.13	2.46 ± 1.29	3.57 ± 0.81*
5	0.13 ± 0.04^a^	0.00 ± 0.02^a^	6.59 ± 6.67	5.26 ± 4.74
7	0.02 ± 0.02^b^	0.00 ± 0.05^b^	3.62 ± 5.37	2.69 ± 3.34


*^a^Daily growth rates calculated from day 5 to day 7.*

^b^Daily growth rates calculated from day 7 to day 9.

*Statistically significantly different from 1 (*p* < 0.05; ANOVA).

In the diel cycle on days 4 to 5, there was an apparent rhythm in Rubisco abundance (measured every 4 h) in the P-replete group associated with the cell cycle and photoperiod (**Figures 6C,D**). It was the lowest at the onset of the dark period (h8), coincident with the active cell division (**Figure 4A**), and increased at the beginning of the light period. In the P-deprived condition, Rubisco abundance not only appeared to be lower but also lack the diel rhythm. However, similar to the case of *F*v/*F*m ratio, large variations occurred among the triplicates, especially in the P-replete group, which made the differences between the two treatments not significant in most cases (**Table 2**).

**Table 2:** Ratios of Rubisco abundance (normalized to GAPDH and per cell) in P-replete group to that in the P-deprived group throughout the diel cycle (mean ± standard deviation).

Hour	Rubisco/GAPDH	Rubisco per cell
		
	P-replete/P-deprived	P-replete/P-deprived
0	4.49 ± 0.07^∗^	2.94 ± 0.62^∗^
4	14.37 ± 20.00	1.55 ± 2.06
8	0.48 ± 0.12^∗^	0.63 ± 0.64
12	2.63 ± 1.80	2.47 ± 2.10
16	3.18 ± 2.97	7.21 ± 8.97
20	20.35 ± 17.33	46.96 ± 57.47
24	3.57 ± 1.14	2.84 ± 2.74


*Gray shade indicates the dark period. ^∗^Statistically significantly different from 1 (*p* < 0.05; ANOVA).*

## Discussion

### The Depression of Population Growth and Stagnation of Cell Cycle Progression under P Deficiency

Similar to previous studies on other species of dinoflagellates (e.g., Zhang et al., 2014; Li et al., 2015), *A. carterae* under P-replete condition exhibited a higher growth rate and a higher cell yield than the P-deprived group. Based on the clear effect of P deficiency, the initial population growth in the P-deprived group we observed must have been supported by P stored inside cells from earlier P-replete growth condition. The differential growth rates corresponded with the active cell cycle progression in the P-replete group and cell cycle arrest at G1 in the P-deprived group. Furthermore, our observation indicates a unique temporal pattern of cell cycle progression in *A. carterae* under the P-replete condition. In all phytoplankton, the cell cycle is entrained by the light and dark cycle (for review see Vaulot, 1994). Dinoflagellates usually exhibit a rhythm in which DNA synthesis occurs at the beginning of the dark period while cytokinesis takes place between the end of the dark period and the beginning of the light period (Swift and Durbin, 1972; Zhuang et al., 2013; Zhang et al., 2014; Li et al., 2015). However, some dinoflagellate species deviates from this general pattern. Grown under a 14:10 light/dark cycle, *P. donghaiense* synthesizes DNA mainly in the middle of the dark period, undergoes mitosis near the onset of the light period and cytokinesis mainly in the first 2 h of the light period (Shi et al., 2013; Li et al., 2015). It has been shown that *A. operculatum* cells synthesize DNA in the middle of the light period and finish mitosis during the whole dark period in a 16:8 light/dark cycle (Leighfield and Van Dolah, 2001). The field population of *K. brevis* was shown to commence DNA synthesis in the light period and complete mitosis at the end of the dark period both in 16:8 and 12:12 light/dark cycles (Van Dolah and Leighfield, 1999). Different from these species, *A. carterae* cultures grown under the P-replete condition and 14:10 light/dark cycle in the present study exhibited the most active DNA synthesis in the middle of the light period following cell size increase, mitosis at dusk, and cytokinesis in the mid dark period. The comparison of our present observation with the previous others indicates that the circadian rhythms of the cell cycle differ among different dinoflagellate species and are influenced by the light dark regime.

Our results also showed the cells of *A. carterae* under the P-deficient condition was arrested in the G1 phase, therefore the circadian rhythm disappeared. This is similar to several other dinoflagellates that have been investigated, including *P. donghaiense* (Li et al., 2015), *A. pacificum* (Zhang et al., 2014), and *K. mikimotoi* (Lei and Lu, 2011). Phosphorus is necessary for synthesis of ribonucleic acids and nucleotides and phosphorylation of proteins (Fisher et al., 2012), the latter of which is the major mechanism by which the cell cycle- regulating cyclin-CDK complexes are activated (Piggott et al., 1982; Nasmyth, 1993; for review see Arellano and Moreno, 1997). There are two major check points in eukaryotic organisms, which monitor the cells entering the S phase from the G1 phase and the M phase from the G2 phase, respectively (Hartwell et al., 1974; Nurse, 1994). The deficiency of phosphorus may have prevented the cells from photosynthetically fixing enough organic carbon, synthesizing enough vital cellular components such as DNA or proteins, or phosphorylating the checkpoint proteins, thus blocking the cells from progressing into S and subsequent phases. To determine which of these possibilities is more likely to be at play, we have analyzed several cellular parameters, which will be discussed below.

### Impacts of P Deficiency on Photosynthesis and Rubisco Abundance

As a major nutrient, the deficiency of phosphorus can impact photosynthesis as well as other cellular activities. The maximum quantum yield of PS II photochemistry (*F*v/*F*m ratio), an indicator of physiological and photosynthetic status (for review see Roháček and Barták, 1999; Parkhill et al., 2001; Herlory et al., 2007), has been reported to reduce under nutrient stress. Declined *F*v/*F*m ratios have been reported in the marine diatoms *P. tricornutum* (Lin et al., 2013), *T. weissflogii* (Liu et al., 2011), and the green algae *Sphaerocystis* and *Scenedesmus* (Beardall et al., 2001) under P limitation. The reduction of *F*v/*F*m ratio also occurs in the green alga *D. tertiolecta* under nitrogen limitation as well as P limitation (Geider et al., 1998). In the present study, our results showed that the photosynthetic capacity of *A. carterae* did not show consistent and statistically significant difference between the two P treatment groups. This suggests that overall photosynthetic efficiency of *A. carterae* was not significantly inhibited by P limitation.

Rubisco abundance has been shown to be down-regulated in response to P deficiency in various phytoplankton species. In the haptophyte *E. huxleyi*, for instance, Rubisco abundance decreased under P limitation (Losh et al., 2013). Rubisco transcription in the marine diatom *S. costatum* displayed significant correlations with growth rate, photosynthetic rate and phosphate concentrations in the medium (Liu et al., 2013). However, it has been shown that *P. tricornutum* Rubisco is upregulated, as detected by proteomic analyses (Feng et al., 2015), or remains constant at the normal level under P starvation (Geider et al., 1993). To our knowledge, dinoflagellate Rubisco has not been studied previously under P deficiency conditions. In the present study, no significant reduction in Rubisco abundance was detected in *A. carterae* in response to P limitation. This indicates that carbon-fixing potential was not drastically decreased in the P-deprived group, hence consistent with the increased C content we observed.

Generally, Rubisco gene transcription is regulated by the light-dark cycle (Chow and Tabita, 1994; Paul et al., 2000; Shi et al., 2013). A previous study documented a strong diel rhythm in Rubisco abundance in the dinoflagellate *P. donghaiense* (Shi et al., 2013). In the present study, we found a circadian rhythm in Rubisco abundance in the P-replete *A. carterae* cultures despite the large within-treatment-group variations. It seems that in both species Rubisco abundance was lower at the beginning of the dark period, increased in the dark period, and peaked at the beginning of the light period, indicating a general feature that cells prepare for photosynthesis far before daylight starts. The disappearance of the rhythm in Rubisco abundance in the P-depleted group indicated the disruption of endogenous rhythm regulators. Perhaps this should not be a surprise because the circadian rhythm of living organisms is regulated by P-containing signal molecules, such as cAMP, which has been shown to play a role in regulating the diel phasing of dinoflagellate cell cycles (Leighfield and Van Dolah, 2001). Likely, P deficiency has blocked the synthesis of the signals in *A. carterae*.

### Enlarged Cell Size and Increased Cellular Biochemical Contents under P Deficiency

Cells in the P-deprived group of *A. carterae* enlarged their size, as has been observed previously in other dinoflagellate species such as *Heterocapsa* sp. (Latasa and Berdalet, 1994), *Ostreopsis* cf. *ovata* (Vanucci et al., 2012), *A. pacificum* (Zhang et al., 2014), and *P. donghaiense* (Li et al., 2015). The enlarged cell size clearly has resulted from the stalled cell cycle progression, but, as demonstrated in the present study, was also due to the sustained photosynthesis supported by the maintained abundance of Rubisco as discussed above.

Two major models have been proposed to describe the cell division gating mechanism named as “sizer” that the commitment of the cells to divide is triggered by a critical size, and “timer” that cells are committed to mitosis at a specific time (Taheri-Araghi et al., 2015). In the “sizer” paradigm, cells are not allowed to advance into the next stage until the size reaches a threshold (for review see Turner et al., 2012). In the dinoflagellate *A. operculatum*, a study showed that exponentially grown cells were varied in cell size and reached maximum size simultaneously with mitosis, while cells blocked in G1 by inhibitor olomoucine hardly changed cell size over the diel cycle (Leighfield and Van Dolah, 2001). These indicate that cell size is not the absolute determinant of cell cycle progression and G1-arrested cells do not necessarily grow in size.

Consistent with the maintained Rubisco abundance, it is intriguing to find higher chlorophyll *a*, carbon, nitrogen, and protein contents in the P-deprived group. This indicates that under P deficiency, cells stalled in G1 were still able to photosynthesize, fix carbon, and assimilate N nutrient to synthesize proteins, resulting in the accumulation of these cellular and biochemical components. There was significant interdependency (*p* < 0.05) between cell size and these cellular contents (**Supplementary Figure S1**). The enlarged cell size was largely accounted for by protein increase and carbon accumulation. The C: N ratio of the P-limited cells increased over time and exceeded the Redfield ratio of C: N = 106:16 (Redfield, 1958) while it was maintained at the Redfield ratio in the P-replete group. This must have resulted from disproportionately increased storage of carbohydrates or lipids relative to proteins and other N-containing molecules under P limitation, clear evidence of sustained photosynthetic carbon fixation. The increase in chlorophyll *a* and largely unreduced PSII photochemical efficiency and Rubisco abundance must have work synergistically to allow sustained photosynthetic carbon fixation contributing to the accumulation of C and protein (**Supplementary Figure S3**). Under P stress, the chlorophyte *Ankistrodesmus falcatus* also exhibit higher carbohydrate and protein contents as well as total lipid contents, with larger and denser cells (Kilham et al., 1997). *P. tricornutum* was shown to increase carbon content by 3% with the upregulation of photosynthesis and carbon assimilation under P limitation, when cells have acquired the cell size threshold but were not qualified to pass through G1-S checkpoint and synthesize DNA (Yang et al., 2014). All these may represent an evolutionary adaptation in which phytoplankton like *A. carterae* gain large cell sizes with accumulated proteins and carbon (likely carbohydrates) under P deficiency such that they are able to resume cell cycle and proliferation rapidly once phosphorus becomes available again.

## Conclusion

This study documents the impacts of phosphorus deficiency on physiological and molecular status of *A. carterae*. Under P deficiency, population growth rate was repressed and cell cycle progression stalled in G1 phase; photosynthetic capacity was maintained; cell size was increased, along with elevated cellular content of C, N, and proteins, presumably a strategy for rapid resumption to proliferation upon resupply of phosphorous nutrient; and rhythm of Rubisco abundance was repressed. The molecular regulatory cascades underlying the arrest of the cell cycle and the loss of the circadian rhythm in Rubisco abundance, however, remain to be elucidated in the future. Furthermore, a remarkable molecular and biochemical reconfiguration must have occurred in order for the cells to maintain a comparable photosynthetic capacity under P deficiency, which should be investigated as well.

## Author Contributions

All authors listed, have made substantial, direct and intellectual contribution to the work, and approved it for publication. SL designed the study. ML conducted the experiments. XS contributed to the experimental design and data analysis. CG contributed to the performance of the experiments and data analysis. ML, SL, XS, CG wrote the paper.

## Conflict of Interest Statement

The authors declare that the research was conducted in the absence of any commercial or financial relationships that could be construed as a potential conflict of interest.
