# Supplementary material for: Phosphorus Deficiency Inhibits Cell Division But Not Growth in the Dinoflagellate Amphidinium carterae
Source: Front Microbiol. 2016 Jun 1;7:826. doi: 10.3389/fmicb.2016.00826 (PMC4887478; doi:10.3389/fmicb.2016.00826)
Supplement: Supplementary file 1 [file Presentation_1.PDF]

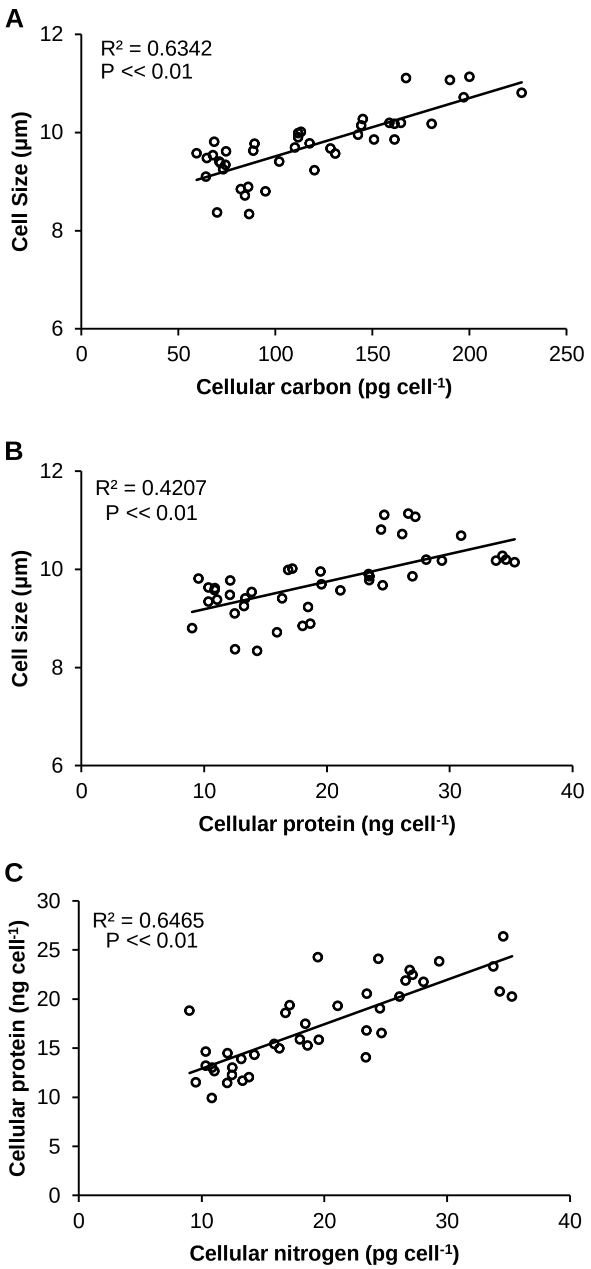

Supplementary Figure S1. Regression analyses between cell size and carbon (A), cell size and cellular protein (B), as well as protein concentration and nitrogen (C).

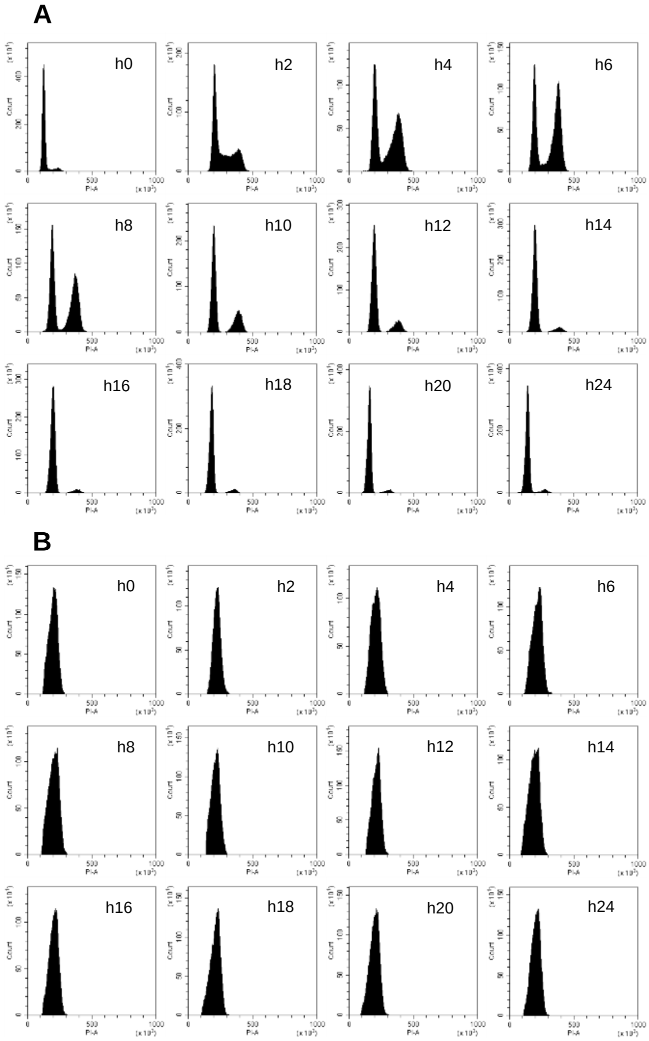

Supplementary Figure S2. Diel temporal progression of flow cytometric DNA profiles in *A. carterae* indicating cell cycle progression in the P-replete group (A) and the lack thereof in the P-deprived group (B).

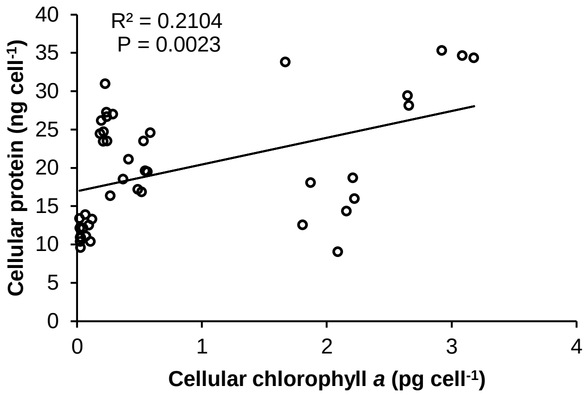

Supplementary Figure S3. Linear correlation between cellular protein and chlorophyll *a* ($p<0.05$).
